# Nursing Interventions Related to the Need for Oxygenation in Severe COVID-19 Disease in Hospitalized Adults: A Retrospective Study

**DOI:** 10.3390/nursrep14040227

**Published:** 2024-10-22

**Authors:** Nicolás Santiago-González, María de Lourdes García-Hernández, Patricia Cruz-Bello, Lorena Chaparro-Díaz, María de Lourdes Rico-González, Yolanda Hernández-Ortega, Jesús Santiago-Abundio

**Affiliations:** 1 Unidad de Proyectos de Investigación en Enfermería del Hospital Regional de Alta Especialidad de Ixtapaluca, Instituto Mexicano de Seguro Social para el Bienestar (IMSS-BIENESTAR), Carretera Federal México-Puebla Km 34.5, Ixtapaluca 56530, Mexico; nicosantiago21@hotmail.com (N.S.-G.); jesus.santiagoab@alumno.buap.mx (J.S.-A.); 2 Facultad de Enfermería y Obstetricia, Universidad Autónoma del Estado de México (UAEMex), Toluca 50000, Mexico; pcruzb@uaemex.mx (P.C.-B.); mlricog@uaemex.mx (M.d.L.R.-G.); yhernandezo@uaemex.mx (Y.H.-O.); 3 Nursing Department, Faculty of Nursing, Universidad Nacional de Colombia, Sede Bogotá, Bogotá 111321, Colombia; olchaparrod@unal.edu.co

**Keywords:** COVID-19, need for oxygenation, nursing care, nursing diagnosis, nursing interventions classification, nursing outcomes classification

## Abstract

COVID-19 affects the respiratory system, reducing the oxygen saturation level, leading to hypoxemia and increasing the metabolic oxygenation need. Objective: To describe the nursing interventions related to the need for oxygenation in hospitalized adults with severe COVID-19 disease in the Intensive Care Unit. Method: This was an observational, retrospective and descriptive study in a population of 2205 patients with a convenience sample of *n* = 430 and based on the North American Nursing Diagnosis Association (NANDA), the Nursing Interventions Classification (NIC) and the Nursing Outcomes Classification (NOC). The analysis was performed with a non-parametric test to determine the association between the nursing interventions and the need for oxygenation. Results: The findings are aimed at improving nursing interventions with statistical associations as follow: oxygen therapy (*p* < 0.000), airway suctioning (*p* < 0.000), airway management (*p* = 0.029), invasive mechanical ventilation (*p* < 0.000) and non-invasive mechanical ventilation (*p* = 0.022). NOC taxonomy expected outcomes in ventilation, 34% (146), alteration in gas exchange, 33.7% (145), and respiratory status, 558.9% (253), were severely compromised. Conclusions: The nursing interventions to maintain the respiratory status are focused on airway care and oxygen therapy in order to increase the oxygen saturation level and decrease the severity of the need for oxygenation.

## 1. Introduction

The Severe Acute Respiratory Syndrome Coronaviruse-2 (SARS-CoV-2) causes COVID-19 disease characterized by lung infection, generating diffuse alveolar damage, inflammation in early stages and hypoperfusion in advanced stages, causing a ventilation–perfusion mismatch, which is the histopathological basis of respiratory distress [1]. acute respiratory distress syndrome (ARDS) presentation consists of dyspnea and oxygen desaturation generating asphyxia sensation, shortness of breath or difficulty breathing, leading to oxygen therapy and respiratory care that help these patients to reduce their need for oxygenation. In Mexico, until 27 December 2022, up to 7,222,611 SARS-CoV-2 cases and 331,030 deaths related to coronavirus were reported, of which 44% were associated with systemic arterial hypertension and 36.6% with diabetes [2].

COVID-19 with chronic comorbidities such as diabetes, systemic arterial hypertension, kidney failure, cardiovascular disease, and cancer increase mortality [3]. In Mexico, we identify that the high prevalence of chronic metabolic diseases leads to a higher risk in comparison to patients without comorbidities; therefore, symptoms and complications are more prevalent in patients with severe COVID-19 [4]. Chronic diseases and smoking are risk factors to fatal outcome and influence in severity [5]; even more, age, physical and cognitive function decay are associated with mortality and increase the adverse outcome risk and nursing intervention complexity [6]. 

ARDS is one of the main manifestations in COVID-19 and frequently requires respiratory support with a medical device. An alternative is to provide oxygen through a high-flow nasal cannula to maintain oxygen saturation levels [7], and the outcome evolution is determined by the oxygen requirement and/or invasive mechanical ventilation management (IMV) [8]. Evidence shows that starting oxygen therapy in early stages improves clinical outcomes [9]. Severe respiratory distress, loss of consciousness and hypoxia are the main indications to place an endotracheal tube; nevertheless, it has to be considered that obesity and senile age are risk factors to a late or difficult extubation [10]. On the other hand, the intubated patient needs airway suctioning, a high-risk intervention due to aerosol generation, increasing the risk of nursing personnel contamination, hence the need for close tracheal suction circuits to avoid alveolar derecruitment, alteration of gas exchange and hypoxia by disconnecting the ventilator circuits [11].

Nursing care must meet basic human needs like normal breathing and oxygenation, thus in patients with severe COVID-19, nursing intervention includes oxygen therapy and airway permeability to maintain SpO2 ≥ 93% and if it is necessary to provide advance life support with IMV [12]. The execution of Nursing Care Plans, drug administration and nutritional support are interventions that can significantly reduce the mortality rate due to severe COVID-19 and improve the recovery/cure rate [13]. Likewise, nursing care and interventions influence patient’s hospital discharge and prevent complications in patients who presented hypoxia [14]. It is worth mentioning that the nursing personnel minimizes SARS-CoV-2 exposure risk and implements interventions to prevent complications in vulnerable patients with chronic diseases [15]; therefore, early case identification is required to establish direct and immediate oxygenation intervention considering that the untreated hypoxia may cause respiratory compromise in patients with poor prognosis [16]. Respiratory distress generates a physiological need to receive oxygen, which can be low, moderate or high, and will depend on the respiratory function indices and clinical outcomes of each patient. 

The theoretical hypothesis is that nursing interventions (assessment of breathing pattern, oxygen therapy, airway suctioning, vital signs monitoring, airway management, changing patient’s position and invasive mechanical ventilation as well as non-invasive) are related to the need for oxygenation in severe COVID-19 disease in adult hospitalized patients.

The objective of this study is to describe the nursing interventions related to the need for oxygenation in severe COVID-19 disease in adult hospitalized patients admitted to the A-ICU.

## 2. Materials and Methods

### 2.1. Study Design and Setting

This was an observational, retrospective and descriptive study with inferential analysis, in which it was determined if there is an association between the studied variables and the nursing interventions. This was analyzed according to the percentage or number of interventions required due to the need for oxygenation in all adult patients hospitalized with severe COVID-19 patients admitted to the Adult Intensive Care Unit (A-ICU) from 1 July 2021 to 30 December 2021 in a public high-specialty hospital in the State of Mexico.

The sampling type was non-probabilistic with convenience sampling; the participants were selected according to the study criteria, resulting in a sample size of 430 patients with severe COVID-19 disease.

### 2.2. Inclusion Criteria

Adult patients with severe COVID-19 that needed oxygenation (low, mild or high) were included. Pregnant women, patients with respiratory disease aside from COVID-19 disease and those whose medical records were incomplete or unavailable were not taken into consideration.

The variable “need for oxygenation” was defined as requiring complementary oxygen therapy administered by any medical device, meeting one or more of the following criteria: (a) use of a medical device to provide oxygen, (b) presence of nasotracheal secretions, (c) breathing pattern alterations and (d) oxygen saturation level less than 90%. 

### 2.3. Data Sources 

The parameters of each variable were only measured once (24 h after the patient’s admission to the A-UCI) and obtained from the electronic clinical record based on the Official Mexican Norm NOM-024-SSA3-2010 and Official Mexican Norm NOM-004-SSA3-2012 [17]. The demographic data, as for the clinical, laboratorial and cabinet results, from all patients are recorded in medical records since the moment of medical admission. No additional or intentional training with the nursing staff was provided due to the continuous academic training received to register all the patient’s data into the clinical record in a standardized manner.

COVID-19 diagnosis was confirmed by real-time polymerase chain reaction (RT-PCR) positive tests for SARS-CoV-2 and severity was evaluated with Kirby’s index, also known as the Fi02/Pa02 ratio (scale that indirectly measures lung injury and determines the degree of hypoxia, which can be mild (>200/<300), moderate (>100/<200) or severe (<100). Comorbidities presented by the patients were evaluated including systemic arterial hypertension, diabetes mellitus, cardiovascular and renal diseases and cancer. 

### 2.4. Data Collection

The instrument used was a data collection sheet structured under the Nursing Care Model [18], which consists of five sections (1) assessment (twenty items), (2) nursing diagnosis (four items), (3) planning (two items), (4) nursing intervention (twenty items) and (5) outcome evaluation (twenty items); each one is described as follows:

1. Evaluation section including demographic data: age (years) and gender (male or female); clinical data: oxygen saturation level less than 90%, presence of nasotracheal secretions and its characteristics (scarce, abundant, fetid, bloody, etc.), capillary blood glucose level (mg/dL) and sedation level (Ramsay’s sedation scale) that evaluates six levels of sedation assigned according to the patient’s response to verbal or painful stimuli: level (1) patient is agitated; (2) patient is co-operating; (3) patient is drowsy but follows orders; (4) patient exhibits response to loud auditory stimulus; (5) patient has a sluggish response to loud auditory stimulus or pain stimuli; and (6) no response. The medical devices to provide oxygen observed include non-invasive mechanical ventilation (NIMV) with continuous positive airway pressure (CPAP), conventional or high-flow nasal prongs and facial masks without reservoirs.
2. Nursing diagnosis is defined as the human responses to health issues and is classified as real or problem-focused diagnosis, risk diagnosis, syndrome diagnosis and health promotion diagnosis according to NANDA-I taxonomy [19].
3. In Nursing Care Plans, the expected outcomes based on the patient’s severity scale status are described and aimed toward reducing the severity. Lastly, the patient’s care plan and the Clinical Practice Guidelines are chosen.
4. The Nursing Interventions Classification (NIC) was used [20]. Nursing interventions are defined as any treatment carried out by the nursing staff for the patients’ benefit, and are related to nursing diagnosis and expected outcome. The taxonomy of these interventions is focused on domain 2, physiological: complex; class k: respiratory management (interventions to promote airway patency and gas exchange). Nursing interventions related to the need for oxygenation, which were recorded by nurses during the pandemic era (2021), were analyzed and listed as follows: patient isolation, airway suctioning, changing patient’s position (proning), infection control, phlebotomy and arterial blood sampling, risk identification, hyperglycemia and hypoglycemia management, mechanical ventilation management (invasive and non-invasive), airway and acute/chronic pain management as well as vital signs monitoring, acid–base balance monitoring, invasive hemodynamic and neurological monitoring, oxygen therapy, precautions to prevent airway aspiration and temperature regulation.
5. Outcome evaluations measured the nursing intervention response and were used as the following five-item Likert-type severity scales according to an evaluation criterion that goes from the most negative to the most positive state mentioned: SeC (severely compromised), SbC (substantially compromised), MC (moderately compromised), MiC (mildly/slightly compromised) and NC (not compromised) established in the Nursing Outcomes Classification taxonomy (NOC) [21].

### 2.5. Data Analysis

Descriptive statistics were used to describe the patient’s demographic and clinical data, as well as the oxygenation medical devices and nursing interventions. The categorical variables were described as the frequency (f) and percentage (%) and the continuous variables with normal distribution were described as the mean and standard deviation (SD). The normality of the continuous variables (age, blood glucose level, oxygen saturation level and fraction of inspired oxygen) was assessed using the Kolmogorov–Smirnov test.

To analyze the need for oxygenation, a score based on the presence or absence of clinical presentation was assigned: use of a medical device to provide oxygen: yes (0) or no (1); airway secretions: 0 (absent) or 1 (present); breathing pattern alteration: 0 (normal breathing pattern) or 1 (altered breathing pattern); oxygen saturation level: 0 (normal range) or 1(hypoxia).

The following scores were also, ranging from zero to four considering the following parameters: 0 (no need for oxygenation), 1 (low need for oxygenation), 2 and 3 (moderate need for oxygenation) and 4 (high need for oxygenation).

The nursing interventions were evaluated using dichotomous answers to each intervention of NIC taxonomy performed by the nursing staff, assigning a score of 0 (not performed) and 1 (performed). 

The statistical analysis related to the interventions with the need for oxygenation were non-parametric tests considering a confidence interval of 95% and a significance level of *p* < 0.05. A Chi-square test (χ^2^ test) of independence between categorical variables was performed. The analysis was carryout using the Statistical Package for the Social Sciences (SPSS v 22) software.

### 2.6. Ethics

This research meets all the statements from the Declaration of Helsinki for medical research in humans [22]. This study was evaluated and approved by the Ethics and Research Committee of the Regional High Specialty Hospital of Ixtapaluca, IMSS-BIENESTAR.

## 3. Results

**Figure 1:**
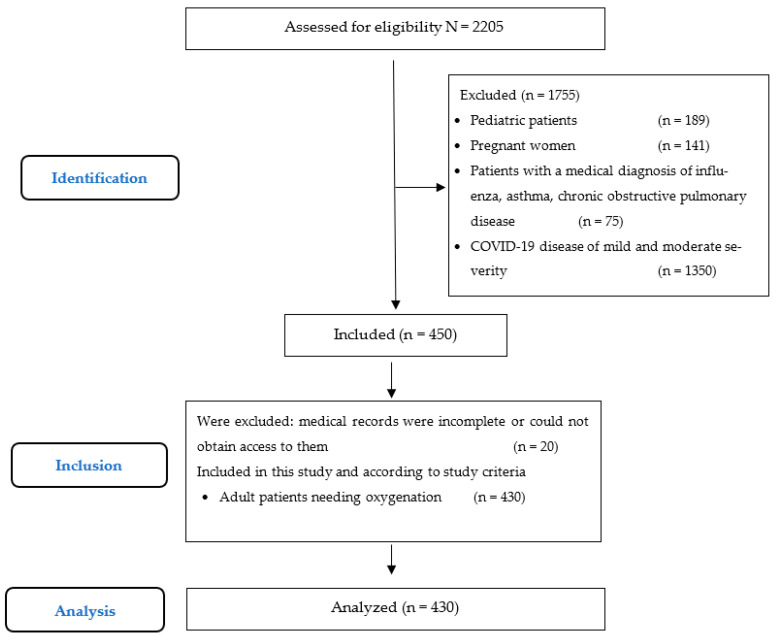
Strengthening the Reporting of Observational Studies in Epidemiology (STROBE) flow chart.

Participants. During this study, 2205 patients were admitted to this hospital with several respiratory pathologies. A total of 1755 were excluded based on the exclusion criteria. A remaining a sample of 430 patients were included in this study, as shown in the flux diagram (Figure 1). Of the population studied, 56% (241) were men, and half of the population were between 45 and 64 years old, with a mean age of 55.2 (SD 15.3) years old. All patients presented one or more comorbidities, mainly diabetes and systemic arterial hypertension in 66% (284). The distribution of chronic diseases is displayed in Table 1.

The need for oxygenation was altered in 92% (395) of all adult hospitalized patients with severe COVID-19. When stratifying the oxygenation need severity, it was noted that 38.6% (164) had a low need, 49.4% (210) had a moderate need and 4% (17) had a high need. At the patient assessment stage, oxygen saturation levels were analyzed with a parameter > 90% in 37.7%, (*p* < 0.000), the minimum oxygen saturation level was 30% and the maximum level was 100% with supplementary oxygen administration. Hypoxia levels were determined as mild in 31.9% (137), moderate in 14.9% (64) and severe in 15.6% (67). The fraction of inspired oxygen (*p* < 0.000) ranged between 15% and 100%, with a mean of 59% (SD 30.07). Only 10.9% (47) from all patients did not require supplementary oxygen administration. The presence of airway secretions was reported in 26% (112) of the population, and characteristics were not described in 8.1% (35), scarce in 6% (26), abundant in 9.1% (39), fetid in 0.5% (2), and bloody in 2.3% (10) of the population. 

Table 2 shows the medical devices used for oxygenation—for example, invasive mechanical ventilation was used in 20.9% (90) and nasal cannula in 28.8% (124) of the population. A total of 53 men (12.3%) and 37 women (8.6%) were intubated. The presence of airway secretions was related to IMV in 77.7% (87) of the cases, with 30.4% (34) as abundant, whereas bloody airway secretions were linked to the intrinsic procedure trauma (airway suctioning). 

Diabetes predominated in the population, leading to blood glucose level testing consequently identifying a mean of 141 mg/dL (SD 83.1) ranging from 39 to 600 mg/dL; a blood glucose level less than 79 mg/dL (hypoglycemia) in 4.7% (20) and 51.9% (223) with a blood glucose level greater than 120 mg/dL (hyperglycemia). Intubated patients and those with psychomotor agitation due to hypoxia were managed with sedation, evaluated with the Ramsay sedation scale, resulting in 19.3% (83) with a score of 1 to 5 points and 16.3% (70) were sedated or unresponsive. The rest of the patients 64.4% (277) did not require evaluation.

The most frequently reported nursing diagnosis in clinical records were: (1) real or problem-focused diagnosis: ineffective respiratory pattern with 54.2% (233) followed by deterioration in gas exchange with 18.6% (80); (2) risk diagnosis: unstable glycaemia in 30.9% (133) and risk of infection in 27.7% (119); (3) syndrome diagnosis and (4) health promotion diagnosis had no record, corresponding to 99.8% (429) and 94.2% (405), respectively.

At the planning stage, the expected outcomes were described and aimed to reduce the severity. Approximately 94.7% (407) of nursing staff used care plans while 92.8% (399) used clinical practice guidelines to support the care provided to the patient.

**Figure 2:**
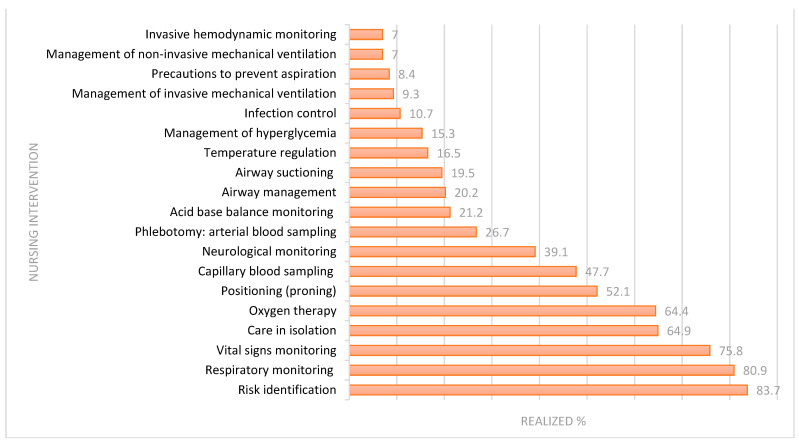
Percentage of nursing interventions in patients who need oxygenation.

The nursing interventions related to the oxygenation need were recorded in the clinical record as shown in Figure 2. The interventions were focused on maintaining a normal respiratory pattern in patients needing oxygenation admitted to the A-UCI.

Table 3 shows the results of the Chi-square test of independence, identifying the nursing interventions with statistical significance (*p* < 0.05) related to the need for oxygenation: airway suctioning (*p* < 0.000), infection control (*p* = 0.033), airway care (*p* = 0.029), acid–base balance monitoring (*p* < 0.000), temperature regulation (*p* = 0.014), arterial blood sampling (*p* < 0.000), invasive mechanical ventilation management (*p* < 0.000) and non-invasive mechanical ventilation management (*p* = 0.022), patient isolation (*p* = 0.011), oxygen therapy (*p* < 0.000), invasive hemodynamic monitoring (*p* < 0.000) and precautions to prevent aspiration (*p* < 0.000). It was identified that the patients provided with the above described nursing interventions had a reduced need for oxygenation.

A severe breathing pattern alteration was identified in 19.9% (81) of patients, so an outcome evaluation was performed according to NOC taxonomy, with several indicators described in Table 4. To describe the NOC outcome, a Likert scale was used, ranging from “non-compromised” to “severely compromised”, identifying a statistical relation between categorical variables (ventilation, integrity of airway mucous membranes, respiratory status and current clinical status acknowledgment).

## 4. Discussion 

Severe COVID-19 disease affects the respiratory pattern and increases the need for oxygenation, requiring the administration of supplementary oxygen either through invasive or non-invasive medical devices. Hospitalized patients in the A-ICU admitted with acute respiratory distress syndrome diagnosis need multiple and continuous respiratory care interventions and close monitoring to meet the oxygenation need.

According to the results, men presented more complications in the respiratory pattern compared to women, and they were also more affected by chronic diseases. These data agree with Mexico National Statistics [2] that indicate systemic arterial hypertension and diabetes as the main comorbidities in all patients, highlighting the recommendations considered as priority in patient care with chronic diseases associated with COVID-19 [23] in order to reduce risk, complications, and lesions as well as lower the high hospitalization costs.

Chronic disease presence, alterations to the need for oxygenation, age and invasive mechanical ventilation management were associated with higher mortality rates; therefore, it is necessary to implement algorithms in patient care so as to mitigate these mortality rate. For example, Bennett et al. demonstrated that chronic diseases in patient are associated with higher clinical severity [24]. 

This study identified an alteration in the need for oxygenation manifested as a low oxygen saturation range, producing mild/moderate/severe hypoxia and thus requiring invasive or non-invasive mechanical ventilation that enables compensating arterial oxygen pressure and the fraction of inspired oxygen as well as the ratio obtained from these two (Pa02/Fi02). In this sense, non-invasive mechanical ventilation management was an alternative that showed good results and decreased mortality rates in the COVID-19 pandemic time [25], highlighting that the nursing interventions should be focused on management and care of non-invasive mechanical ventilation for patient well-being.

Different oxygenation devices were used, including a high-flow nasal cannula, which has been reported to have better results [26], hence nursing intervention involves verifying constant oxygen flow, monitoring respiratory parameters, preventing injures related to high-flow nasal cannula, turning and changing the patient’s position so it favors hemodynamic stability, etc.

An airway response team must perform the management of airway emergencies in the ICU using clinical algorithms and team coordination to favor patient hemodynamic stability [27]. This study observed the frequent use of IMV during the pandemic in severe COVID-19 cases and the mortality rate was high, making effective airway management the main challenge to reduce the incidence of complications and promote clinical quality.

Approximately half of the study population presented hyperglycemia, a fact that must be considered during clinical care since the available evidence points to an association between poor glycemic control and an increase in severity in patients affected by COVID-19 [28], so the mentioned inference can and should be validated by conducting additional research.

The nursing care process is a standardized methodology to evaluate the association between the presence of related/risk factors and clinical decision making to issue nursing diagnoses (NANDA-I) and evaluate the impact of nursing interventions (NIC) and nursing outcomes (NOC) on the patients’ health status—in this case, those who have been admitted with COVID-19 diagnosis [29]—where various diagnostic labels of the taxonomy are described and are consistent in this study. These include gas exchange deterioration, ineffective airway clearance, unstable glycaemia risk and infection risk. It is worth mentioning that an ineffective respiratory pattern was the most frequently diagnosis reported in the A-ICU. 

Critically ill patients due to COVID-19 require significantly more nursing care time in the A-ICU, according to the results obtained by Bruyneel et al., who used the Nursing Activities Score (NAS) to demonstrate that integrated nursing interventions increase care time and exposure, resulting in a greater risk for contracting COVID-19 [30]. This study observed a frequency rise in nursing interventions during critically ill patient care due to COIVD-19, specifically interventions aimed to maintain effective breathing. Therefore, interventions provided by the nursing staff are indispensable to reduce the risk of exposure and work overload in cases that require advanced airway management.

NOC indicators help to evaluate the progressive care status during hospitalization to determine care evolution. The nursing interventions that favored the respiratory status were airway suctioning, infection control, airway management, acid–base balance monitoring, temperature regulation, mechanical ventilation (invasive and non-invasive), patient isolation, oxygen therapy, position changes (proning), invasive hemodynamic monitoring and precautions to prevent aspiration. That said, it is necessary an integrate care and links to a multidisciplinary healthcare team; according to Zeydi et al., nursing staff play a critical role in patient care due to COVID-19, providing quality health care based on experience and clinical research to decrease morbidity and mortality rates [31]. 

There are implications for practice and future research. It is subjective to evaluate the expected outcomes in the study population, despite having validated Likert-type scales and taxonomies, since the physiological response might be similar although the person’s needs, coping strategies, severity and age, physiological mechanisms, pharmacodynamics, etc., influence the result. Therefore, it is suggested to conduct more studies involving nursing outcomes of patients hospitalized due to COVID-19 in the A-ICU.

## 5. Limits 

The main strength of this study is that the analysis referred to nursing interventions provided by the nursing staff in the A-ICU during the acute phase of the COVID-19 pandemic. Limitations include that this is a retrospective study, it was only carried out in a tertiary-care-level center, did not include a randomized sample and the findings cannot be generalized to all severe COVID-19 disease patients. In addition, the epidemiological restriction in that period limited the ability to perform a previous clinical verification exercise to carry out a prospective study.

## 6. Conclusions 

The nursing interventions related to the need for oxygenation in severe COVID-19 disease in hospitalized adults were airway suctioning, infection control, airway management, acid–base balance monitoring, temperature regulation, invasive and non-invasive mechanical ventilation management, patient isolation, oxygen therapy, invasive hemodynamic monitoring and precautions to avoid aspiration. The patients provided with this nursing intervention were less compromised in terms of the respiratory pattern status associated with a low need for oxygenation. In addition, an improvement in clinical status was identified from severely compromised to moderately compromised according to the Likert score in NOC taxonomy. Chronic comorbidities and COVID-19 disease symptom management as well as nursing interventions must be aimed to prevent complications, meet physiological needs and maintain an effective respiratory pattern. 

## Figures and Tables

**Table 1:** Percentage of patients with chronic disease.

COVID-19 Disease with Chronic Disease	Total	Gender	
	*n* (%)	Female *n* (%)	Male *n* (%)
Diabetes mellitus	156 (36.3%)	67 (15.6%)	89 (20.7%)
Diabetes and arterial hypertension (simultaneous)	66 (15.3%)	39 (9.1%)	27 (6.3%)
Systemic arterial hypertension	62 (14.4%)	29 (6.7%)	33 (7.7%)
Cardiovascular disease	59 (13.7%)	25 (5.8%)	34 (7.9%)
Chronic kidney disease	27 (6.3%)	4 (0.9%)	23 (5.3%)
Oncological disease	27 (6.3%)	15 (3.5%)	12 (2.8%)
Other	33 (7.7%)	10 (2.3%)	23 (5.3%)
Total	430 (100.0%)	189 (44.0%)	241 (56.0%)

Source: Own preparation.

**Table 2:** Percentage of hospitalized patients with invasive and non-invasive oxygenation devices.

Oxygenation Devices	Gender		Total *n* (%)
	Female *n* (%)	Male *n* (%)	
Nasal prongs	54 (12.6%)	70 (16.3%)	124 (28.8%)
Invasive mechanical ventilation	37 (8.6%)	53 (12.3%)	90 (20.9%)
Reservoir Mask	33 (7.7%)	44 (10.2%)	77 (17.9%)
High-Flow Nasal Prong	31 (7.2%)	36 (8.4%)	67 (15.6%)
None	23 (5.3%)	24 (5.6%)	47 (10.9%)
Oxygen mask	7 (1.6%)	12 (2.8%)	19 (4.4%)
Tracheostomy (Invasive)	2 (0.5%)	2 (0.5%)	4 (0.9%)
Non invasive mechanical ventilation	2 (0.5%)	0 (0.0%)	2 (0.5%)
Total	189 (44.0%)	241 (56.0%)	430 (100.0%)

**Table 3:** Association of nursing interventions with the need for oxygenation.

Nursing Interventions	Total *n* = 430 (100.0%)	Oxigenation Need				χ^2^	
		No Need *n* (%)	Low *n* (%)	Moderate *n* (%)	High *n* (%)	Value	*p* < 0.05
Airway suctioning	84 (19.5%)	1 (0.2%)	3 (0.7%)	68 (15.8%)	12 (2.8%)	99.47	0.000
Infection control	46 (10.7%)	1 (0.2%)	22 (5.1%)	20 (4.7%)	3 (0.7%)	13.74	0.033
Airway management	87 (20.2%)	1 (0.2%)	31 (7.2%)	53 (12.3%)	2 (0.5%)	14.07	0.029
Acid-Base Balance Monitoring	91 (21.2%)	3 (0.7%)	14 (3.3%)	65 (15.1%)	9 (2.1%)	50.14	0.000
Temperature regulation	71 (16.5%)	4 (0.9%)	19 (4.4%)	43 (10.0%)	5 (1.2%)	15.96	0.014
Phlebotomy: Arterial blood sample	115 (26.7%)	7 (1.6%)	13 (3.0%)	88 (20.5%)	7 (1.6%)	67.06	0.000
Management of invasive mechanical ventilation	40 (9.3%)	0 (0.0%)	2 (0.5%)	33 (7.7%)	5 (1.2%)	45.43	0.000
Management of non-invasive mechanical ventilation	30 (7.0%)	0 (0.0%)	17 (4.0%)	13 (3.0%)	0 (0.0%)	14.73	0.022
Isolation	278 (64.9%)	26 (6.0%)	110 (25.6%)	130 (30.2%)	13 (3.0%)	16.66	0.011
Oxygen therapy	277 (64.4%)	6 (1.4%)	143 (33.3%)	117 (27.2%)	11 (2.6%)	73.48	0.000
Invasive hemodynamic monitoring	30 (7.0%)	0 (0.0%)	3 (0.7%)	24 (5.6%)	3 (0.7%)	28.19	0.000
Precautions to prevent aspiration	36 (8.4%)	0 (0.0%)	7 (1.6%)	24 (5.6%)	5 (1.2%)	29.78	0.000

Source: Own preparation.

**Table 4:** Patients with severe breathing pattern alteration evaluated with NOC taxonomy.

NOC Indicators	NR *n* (%)	SeC *n* (%)	SuC *n* (%)	MoD *n* (%)	MiC *n* (%)	NC *n* (%)	χ^2^	*p* < 0.05
Ventilation	50 (61.7%)	7 (8.6%)	7 (8.6%)	13 (16.0%)	3 (3.7%)	1 (1.2%)	22.49	0.001
Selfconcept	77 (95.1%)	0 (0.0%)	2 (2.5%)	0 (0.0%)	1 (1.2%)	1 (1.2%)	9.45	0.092
Infectious risk control	65 (80.2%)	0 (0.0%)	4 (4.9%)	5 (6.2%)	6 (7.4%)	1 (1.2%)	6.48	0.232
Gas exchange	48 (59.3%)	1 (1.2%)	9 (11.1%)	12 (14.8%)	9 (11.1%)	2 (2.5%)	7.309	0.293
Airway patency	61 (75.3%)	0 (0.0%)	4 (4.9%)	9 (11.1%)	6 (7.4%)	1 (1.2%)	9.27	0.159
Blood glucose level	53 (65.4%)	1 (1.2%)	10 (12.3%)	9 (11.1%)	7 (8.6%)	1 (1.2%)	13.48	0.036
Tissue integrity: skin	54 (66.7%)	0 (0.0%)	2 (2.5%)	18 (22.2%)	6 (7.4%)	1 (1.2%)	23.20	0.001
Acceptance: health status	61 (75.3%)	1 (1.2%)	6 (7.4%)	6 (7.4%)	4 (4.9%)	3 (3.7%)	13.81	0.032
Respiratory status	36 (44.4%)	2 (2.5%)	15 (18.5%)	17 (21.0%)	10 (12.3%)	1 (1.2%)	21.36	0.002
Mechanical ventilation	75 (92.6%)	0 (0.0%)	2 (2.5%)	3 (3.7%)	0 (0.0%)	1 (1.2%)	11.78	0.067
Preventing Aspiration	75 (92.6%)	0 (0.0%)	2 (2.5%)	1 (1.2%)	2 (2.5%)	1 (1.2%)	7.53	0.275

Note: the patient’s health status was evaluated using the Likert scale (NOC): NR = No record, SeC = Seriously compromised, SuC = Substantially compromised, MoD = Moderately compromised, MiC = Mildly compromised, and NC = Not compromised.

## Data Availability

We are happy to share the data, so please request it from the corresponding author.

## Public Involvement Statement

There was no public involvement in any aspect of this research.

## Guidelines and Standards Statement

This study was realized according to the Strengthening the Reporting of Observational Studies in Epidemiology (STROBE) statement [32].
